# The Drought Tolerance Function and Tanscriptional Regulation of *GhAPX7* in *Gossypium hirsutum*

**DOI:** 10.3390/plants13152032

**Published:** 2024-07-24

**Authors:** Tingwei Wang, Quanjia Chen, Yaping Guo, Wenju Gao, Hu Zhang, Duolu Li, Shiwei Geng, Yuxiang Wang, Jieyin Zhao, Jincheng Fu, Yilei Long, Pengfei Liu, Yanying Qu, Qin Chen

**Affiliations:** Engineering Research Centre of Cotton, Ministry of Education, College of Agriculture, Xinjiang Agricultural University, 311 Nongda East Road, Urumqi 830052, China; 15756192290@163.com (T.W.); chqjia@126.com (Q.C.); guoyaping1217@163.com (Y.G.); gaowenju120@gmail.com (W.G.); 18140750187@163.com (H.Z.); lf10160914@163.com (D.L.); gengshiwei20201231@163.com (S.G.); wangyx2023888@163.com (Y.W.); cottonzjy@126.com (J.Z.); fjc5202023@163.com (J.F.); longyilei2021@163.com (Y.L.); lpfei007@163.com (P.L.)

**Keywords:** *GhAPX7*, virus-induced gene silencing, molecular mechanisms of drought resistance, transcriptome analysis

## Abstract

Drought stress significantly affects the growth, development, and yield of cotton, triggering the response of multiple genes. Among them, ascorbate peroxidase (APX) is one of the important antioxidant enzymes in the metabolism of reactive oxygen species in plants, and APX enhances the ability of plants to resist oxidation, thus increasing plant stress tolerance. Therefore, enhancing the activity of APX in cells is crucial to improving plant stress resistance. Previous studies have isolated differentially expressed proteins under drought stress (*GhAPX7*) in drought-resistant (KK1543) and drought-sensitive (XLZ26) plants. Thus, this study analyzed the expression patterns of *GhAPX7* in different cotton tissues to verify the drought resistance function of *GhAPX7* and explore its regulatory pathways. *GhAPX7* had the highest expression in cotton leaves, which significantly increased under drought stress, suggesting that *GhAPX7* is essential for improving antioxidant capacity and enzyme activities in cotton. *GhAPX7* silencing indirectly affects pronounced leaf yellowing and wilting in drought-resistant and drought-sensitive plants under drought stress. Malondialdehyde (MDA) content was significantly increased and chlorophyll and proline content and APX enzyme activity were generally decreased in silenced plants compared to the control. This result indicates that *GhAPX7* may improve drought resistance by influencing the contents of MDA, chlorophyll, proline, and APX enzyme activity through increased expression levels. Transcriptome analysis revealed that the drought-related differentially expressed genes between the control and treated groups enriched plant hormone signal transduction, MAPK signaling, and plant–pathogen interaction pathways. Therefore, the decreased expression of *GhAPX7* significantly affects the expression levels of genes in these three pathways, reducing drought resistance in plants. This study provides insights into the molecular mechanisms of *GhAPX7* and its role in drought resistance and lays a foundation for further research on the molecular mechanisms of response to drought stress in cotton.

## 1. Introduction

Drought poses a severe global challenge, significantly impacting plants throughout their life cycle by imposing various abiotic stresses. With the ongoing effects of climate change, the frequency and duration of droughts are increasing alarmingly, causing substantial agricultural losses annually [1]. In China, agricultural water resources are critically scarce, particularly in the cotton-growing regions of Xinjiang. Drought is among the most harmful abiotic stresses affecting cotton growth and yield. Thus, understanding the molecular mechanisms of response to drought stress in cotton is crucial [2]. Multiple genes regulate drought response in cotton. However, the complexity of drought-related traits makes the molecular mechanisms underlying drought resistance in cotton unclear. Therefore, identifying drought-resistant genes and elucidating the molecular mechanisms of drought resistance are essential for improving cotton yield and quality, contributing to the sustainable development of the cotton industry.

A key strategy to increase plant stress resistance is enhancing the activity of antioxidant enzymes and antioxidant metabolism in plant cells. The response of APX (ascorbate peroxidase) to various environmental factors varies among plants, indicating that the physiological basis and environmental sensitivity of *APX* may differ among plant species. This difference suggests *APX* as a physiological and biochemical indicator in crop stress resistance breeding. Numerous studies have shown that overexpressing *APX* can enhance the resistance of transgenic plants to various stresses. The *APX* gene was initially discovered while investigating new catalytic products and scavenging mechanisms of H_2_O_2_ [3]. In 1981, the APX enzyme was formally identified as ascorbate peroxidase, and the molecular and enzymatic characteristics of *APX* were extensively explored [3]. Early research primarily focused on Arabidopsis at the molecular level, followed by studies on economically important crops such as soybeans [4]. Researchers have investigated the cloning and expression characteristics, genetic transformation, and functional identification of *APX* genes. Recent research on *APX* in cotton has mainly concentrated on the cloning of fiber-specific genes and the regulatory mechanisms of *APX* in cotton fiber development [5]. Exogenous thylakoid membrane-bound *APX* can enhance the PSII (Photosynthesis II) photochemical activity and antioxidant capacity of transgenic cotton [6]. However, research on *APX* in stress resistance and its molecular mechanisms remains relatively scarce. Therefore, studying the molecular mechanisms of drought resistance in upland cotton genes is significant.

In plants, virus-induced gene silencing (VIGS) can be used for reverse and forward genetic screening [7]. Unlike traditional gene function validation methods, VIGS offers several irreplaceable advantages. Firstly, VIGS can produce phenotypes in the current generation with a shorter transformation time within the plant, allowing for rapid validation of target gene functions [8]. Secondly, the coding region has a wide selection range because the silenced target fragment length typically ranges from 300 to 500 bp. The VIGS mechanism [9] involves post-transcriptional silencing caused by specific mRNA degradation in the cytoplasm [10]. Finally, gene function analysis is conducted based on the phenotype of the silenced plants [11]. TRV-mediated VIGS technology has been widely applied in plants [12], making it a research hotspot for exploring gene function and breeding new varieties [10].

Researchers have identified *GhWRKY59*, a key transcription factor regulating drought stress response in cotton, by integrating whole-genome RNA sequencing (RNA-Seq), VIGS functional loss screening, and comprehensive biochemical analysis [13]. *GhWRKY59* is a TUBBY-like protein (TULP) whose role in stress responses in cotton is scarcely reported. Silencing *GhVHA-A* via VIGS reduced drought resistance in cotton, decreasing the chlorophyll content and antioxidant enzyme activity. Thus, *GhVHA-A* is a candidate gene for enhancing drought resistance in cotton [14]. Drought stress activates *GhTULP30* [15]. Thus, silencing *GhTULP30* in cotton through VIGS reduced the stomatal closure rate and decreased stomatal width and length under drought stress, providing a reference for further exploring the role of *TULP* in cotton [15].

VIGS-mediated silencing of two Ca^2+^-related genes, *GhECA1* and *GhCNGC4*, reduced drought resistance in cotton and lowered the m6A concentration in the silenced genes, revealing a new post-transcriptional modification mechanism that influences drought response in cotton. This post-transcriptional modification mediates m6A methylation of targeted transcripts in the ABA and Ca^2+^ signaling pathways [16]. Additionally, VIGS-based silencing of *GhGAPDH9* caused noticeable wilting or complete desiccation of plant leaves under drought stress, significantly increased the malondialdehyde (MDA) content, and decreased the proline (Pro) and chlorophyll (Chl) contents. Therefore, *GhGAPDH9* positively regulates drought resistance in cotton [17]. Transcriptomics can be employed to unravel the roles of *GhGAPDH9* in cotton.

Currently, transcriptomics can reflect the transcriptional and regulatory patterns of all genes in specific cells, tissues, or organisms at different developmental stages and under various stress conditions. This technique allows candidate gene identification and the elucidation of interactions between different regulatory pathways [18]. For instance, Liu Ruidan et al. [19] showed that drought significantly up-regulated the *GhMYB102* gene in *Gossypium hirsutum Linn.* during the early stages of drought stress. Transcriptomics also unraveled multiple drought response genes and pathways in non-model plants such as Populus euphratica [20], cotton [21], and tea [22]. Transcriptome analysis also showed that the scavenging mechanisms of reactive oxygen species (ROS) associated with iron toxicity and RNA-binding proteins positively regulated drought stress in rice [23,24]. Wang et al. identified a drought-induced *WRKY* gene, *ZmWRKY106*, from the drought transcriptome sequence data of maize [25]. An RNA-seq of field-grown cotton under natural rainfall and well-watered conditions identified 1530 differentially expressed transcripts commonly expressed in the root tissues under full irrigation and drought stress [26]. Furthermore, the RNA-seq transcripts of four wild quinoa varieties under different water treatments identified 462 common differentially expressed genes (DEGs) within the same variety under different drought treatments and 27 common DEGs across treatments and varieties [27]. In salt-treated *Nicotiana alata*, transcriptomics revealed that ROS scavenging is one important defense mechanism for salt stress in *Nicotiana alata* [28].

Thus, this study utilized VIGS to examine drought-related physiological indicators and expression changes in drought-related genes, validating the drought resistance function of *GhAPX7*. An RNA-seq analysis revealed that *GhAPX7* plays a crucial role in the plant hormone signal transduction pathway, providing preliminary insights into the molecular mechanisms by which *GhAPX7* operates in the drought stress response of cotton.

## 2. Results

### 2.1. RT-qPCR Analysis of GhAPX7

**Figure 1:**
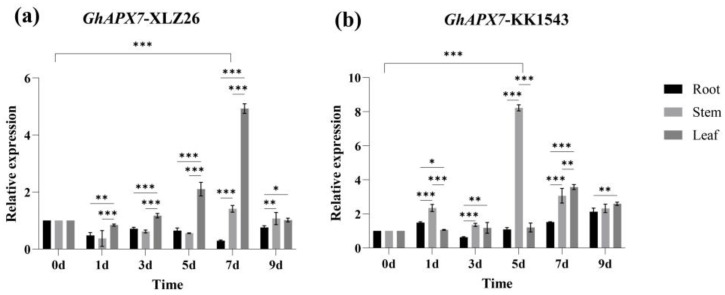
The *GhAPX7* gene expression patterns. (**a**) Tissue-specific expression of *GhAPX7* in XLZ26. (**b**) Tissue-specific expression of *GhAPX7* in KK1543. Statistical analysis was conducted using a one-way analysis of variance (ANOVA). * represents *p* < 0.05, indicating significant differences; ** represents *p* < 0.01, indicating highly significant differences; and *** represents *p* < 0.001, indicating extremely significant differences.

The relative expression level fluctuated in the drought-sensitive material XLZ26, with an overall decreasing trend. Tissue specificity analysis showed that *GhAPX7* expression initially decreased in the roots, then increased, followed by another decrease. The relative expression was highest on the 9th day of natural drought stress, significantly different from that during the normal treatment phase. In stems, the expression initially decreased, then increased, followed by another decrease. The relative expression was highest on the 7th day of natural drought stress, significantly different from that during the normal treatment. In leaves, the expression initially decreased, then increased, followed by another decrease. The relative expression level was highest on the 7th day of natural drought stress, significantly different from that during the normal treatment (Figure 1a).

Figure 1 shows the RT-qPCR results of *GhAPX7* expression under drought stress. Drought stress increased *GhAPX7* expression in the drought-resistant material, KK1543. Tissue-specific analysis showed that *GhAPX7* expression in roots and stems initially increased and then decreased, followed by another increase. The relative expression was maximum on the 9th day of natural drought stress, significantly different from the relative expression during the normal treatment. In stems, the relative expression was highest on the 5th day of natural drought stress, significantly different from that during the normal treatment. In leaves, *GhAPX7* expression initially increased, then decreased. The relative expression level was highest on the 7th day of natural drought stress, significantly different from that during the normal treatment (Figure 1b).

These results suggest that drought stress affects *GhAPX7* expression and that *GhAPX7* expression in plants with different resistances is tissue-specific.

### 2.2. Analysis of GhAPX7 Gene Silencing

#### 2.2.1. Silencing of *GhAPX7* Gene

**Figure 2:**
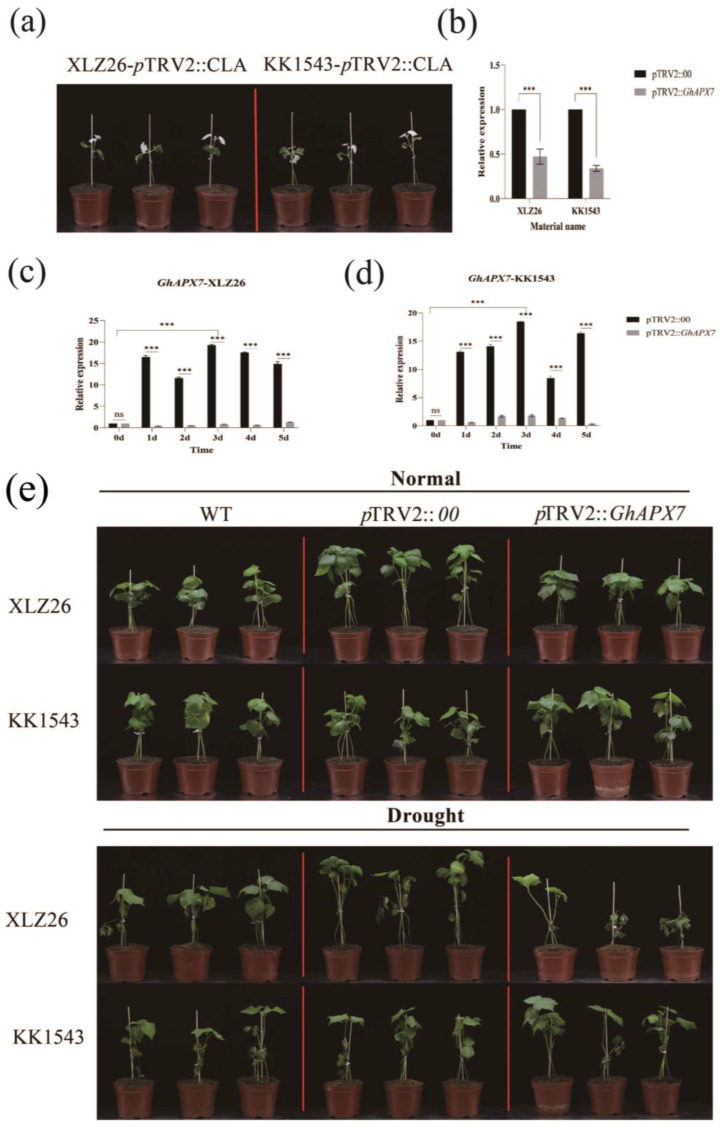
VIGS experiment with *GhAPX7*. (**a**) Whitening phenotype of pTRV2::CLA in XLZ26 and KK1543; (**b**) silencing efficiency detection using one-way analysis of variance (ANOVA); (**c**,**d**) *GhAPX7* expression in pTRV2::00 plants after drought stress; and (**e**) phenotypic comparison between pTRV2::00 and pTRV2::*GhAPX7* materials under normal watering and soil moisture content below 10% for 3 days. Statistical analysis was performed using a two-way analysis of variance (ANOVA); *** represents *p* < 0.001, indicating significant differences.

Figure 2a is an albino phenotype based on *GhAPX7* gene silencing in both XLZ26 and KK1543 parental material. For the calculation of silencing efficiency, the relative expression was calculated using pTRV2::00 plants as controls (Figure 2b). In KK1543 plants, the relative expression of *GhAPX7* was approximately 0.3 in pTRV2::*GhAPX7* plants and approximately 0.4 in pTRV2 in XLZ26 plants. These results indicate a difference in the silencing efficiency of VIGS experiments between the two materials, but gene silencing was successful, ensuring the accuracy of subsequent experiments.

The expression of *GhAPX7* in the drought-sensitive XLZ26 and drought-resistant KK1543 pTRV2::00 parental plants was consistent with the previous RT-qPCR results. PCR analysis was performed using extreme resource materials. During drought stress, *GhAPX7* expression in leaves was significantly higher in drought-resistant than drought-sensitive materials (Figure 2c,d). The results indicate a significant difference in the impact of *GhAPX7* gene silencing on the two materials when the soil moisture content is <10%. The loss of *GhAPX7* gene expression may cause leaf discoloration under drought stress. Under severe stress, all materials exhibited wilting, yellowing, and other dehydration symptoms. In the drought-resistant variety KK1543, pTRV2::*GhAPX7* plants showed an increased degree of wilting and yellowing than pTRV2::00 and WT plants, with significant differences between stem and leaf wilting symptoms. In the drought-sensitive parental XLZ26, pTRV2::*GhAPX7* plants exhibited wilting, yellowing, and drying of leaves compared to pTRV2::00 and WT plants. Additionally, *GhAPX7* silencing exacerbated this effect (see Figure 2e).

#### 2.2.2. Physiological Parameters after *GhAPX7* Gene Silencing

**Figure 3:**
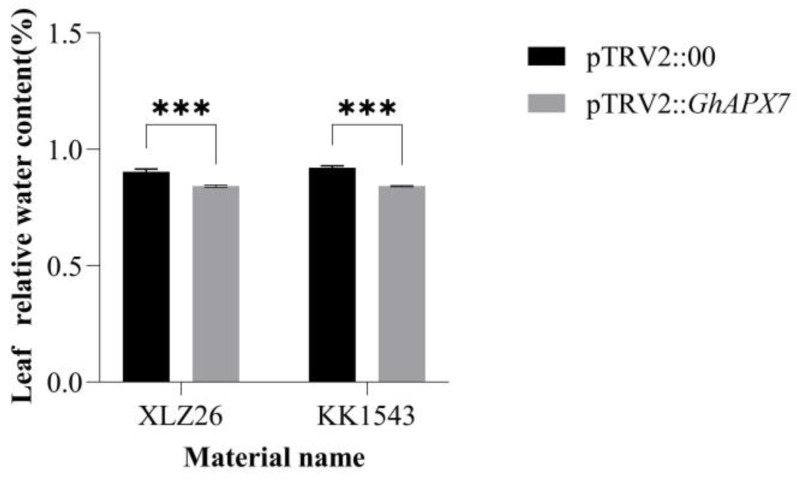
Silencing of relative leaf water content after normal watering and drying in *GhAPX7* cotton. pTRV2:00: control subjects; pTRV2::*GhAPX7*: test group. Silencing efficiency detection using one-way analysis of variance (ANOVA); and *** represents *p* < 0.001, indicating significant differences.

In Figure 3, both the KK1543 and XLZ26 experimental group leaves’ relative water content was lower than the control group leaves’ relative water content and there was significant difference. Also, the relative water content of the leaves of both the control and experimental groups of KK1543 plants was a little higher than that of XLZ26 plants.

**Figure 4:**
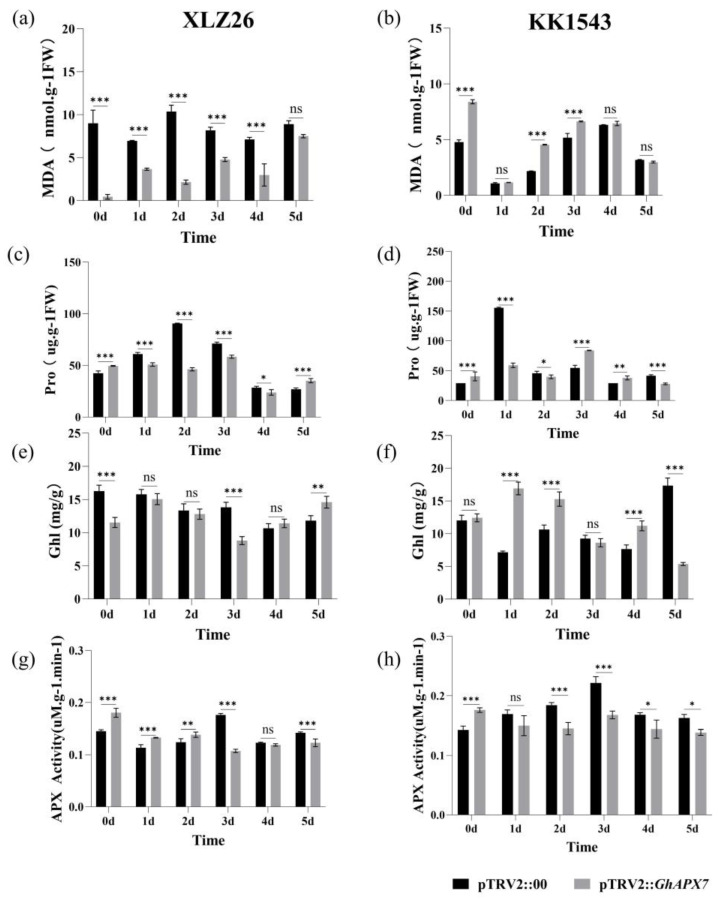
Physiological and biochemical assays of *GhAPX7* VIGS materials. (**a**,**b**), MDA, (**c**,**d**), Pro, (**e**,**f**), Chl, (**g**,**h**), APX, in pTRV2::00 and pTRV2::*GhAPX7* in drought-stressed XLZ26 and KK1543, respectively. Statistical analysis was performed using a two-way analysis of variance (ANOVA). * represents *p* < 0.05, indicating a significant difference; ** represents *p* < 0.01, indicating a highly significant difference; *** represents *p* < 0.001, indicating an extremely significant difference.

Drought stress significantly reduced the MDA content in the receptor materials XLZ26 and KK1543 and pTRV2::*GhAPX7* plants compared to pTRV2::00 plants. Drought stress significantly decreased the proline content in pTRV2::*GhAPX7* plants with XLZ26 and KK1543 as receptor materials. Therefore, silencing *GhAPX7* changes the MDA content in plants consistently with changes under drought stress and demonstrates the role of *GhAPX7* in response to drought stress in cotton (Figure 4a,b). Drought stress significantly reduced the proline content in pTRV2::*GhAPX7* plants with XLZ26 as the receptor material.

In contrast, the proline content in pTRV2::*GhAPX7* plants with KK1543 as the receptor material initially decreased and then increased (Figure 4c,d). Drought stress significantly reduced the chlorophyll content in pTRV2::*GhAPX7* plants with XLZ26 as the receptor material. In contrast, the chlorophyll content in pTRV2::*GhAPX7* plants with KK1543 as the receptor material decreased (Figure 4e,f). Furthermore, drought stress significantly reduced the APX enzyme activity in pTRV2::*GhAPX7* plants with both XLZ26 and KK1543 as receptor materials (Figure 4g,h).

#### 2.2.3. Related Gene Expression after *GhAPX7* Gene Silencing

Drought stress increases the ABA content within plant cells and activates a class of regulatory proteins with a basic leucine zipper structure, such as ABF/ERF1/ABI5. Among them, the transcription factor GhABF2 is a homolog of ABF2, and overexpression of GhABF2 significantly enhances plant drought resistance. Additionally, SOS2 regulates plant responses to stress, particularly in cases of dual stress from shading and salt. Previous studies have shown that GhABF2, ERF1, ABI5, and SOS2 respond significantly to drought stress, and their overexpression can enhance drought resistance in plants.

**Figure 5:**
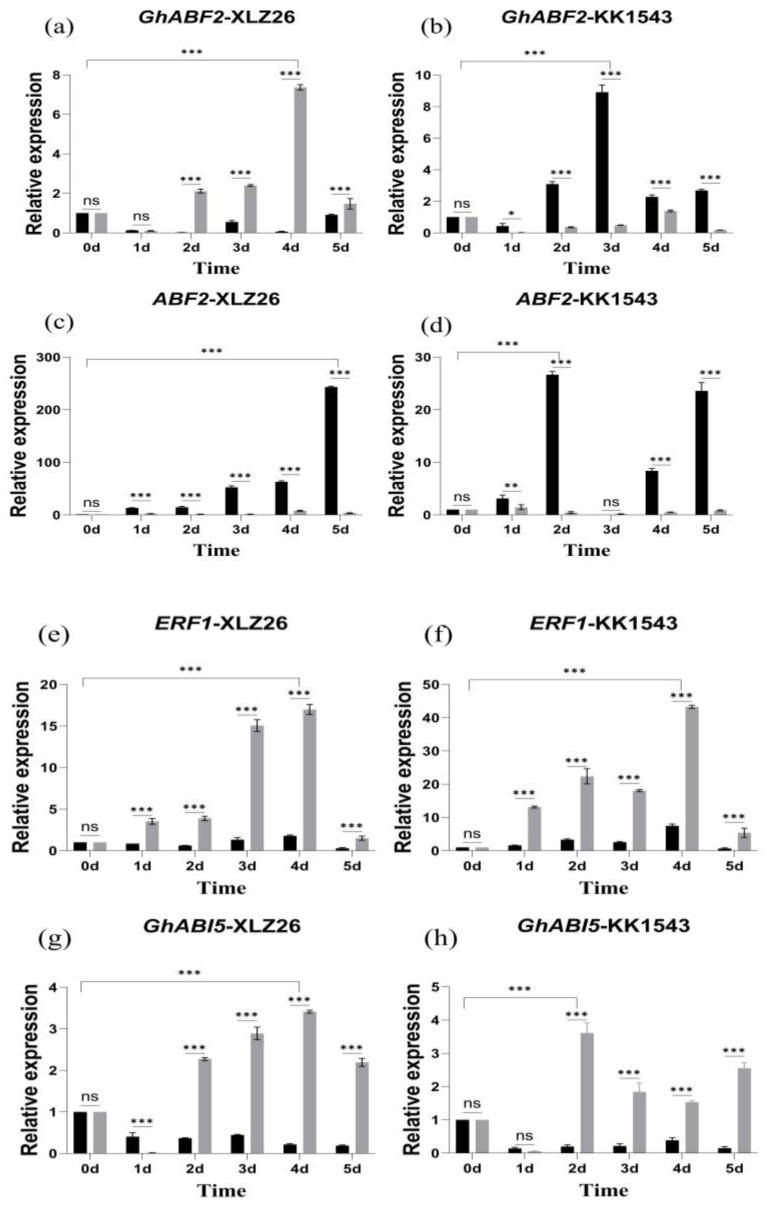

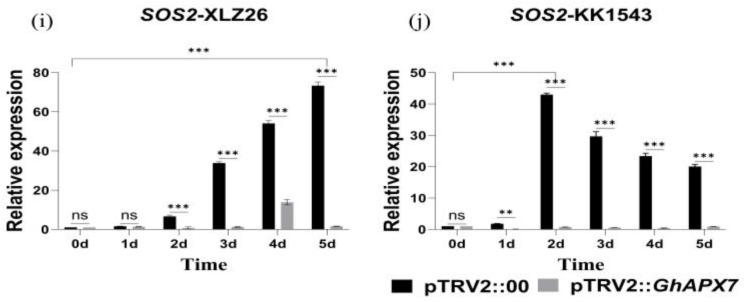
RT-qPCR analysis of drought-responsive marker genes (**a**–**j**) shows the relative expression of GhABF2, ABF2, ERF1, GhABI5, and SOS2 in drought-stressed pTRV2::00 and pTRV2::*GhAPX7* plants of XLZ26 and KK1543. Statistical analysis was performed using a two-way analysis of variance (ANOVA). * indicates *p* < 0.05, indicating significant difference; ** indicates *p* < 0.01, indicating highly significant difference; *** indicates *p* < 0.001, indicating extremely significant difference.

GhABF2 is involved in the ABA-mediated signaling pathways in response to drought and salt stress. Moreover, ABA induces overexpression of GhABF2 to enhance drought resistance in cotton. Experimental results indicate that the expression level of GhABF2 increases relative to the severity of stress. In the drought-sensitive material, Xinluzao26, the relative expression of GhABF2 in pTRV2::*GhAPX7* plants gradually increases compared to pTRV2::00 plants. However, in the drought-resistant KK1543, the relative expression level of GhABF2 in pTRV2::*GhAPX7* plants significantly decreased compared to pTRV2::00 plants (Figure 5a–d).

ERF1 is positioned upstream of the ABA signaling pathway, and its overexpression can inhibit a downstream gene, ABI5 [29], regulating the expression of genes containing the abscisic acid response elements (ABREs) in their promoter regions. As the severity of drought stress increases, ERF1 gradually increases, followed by a decrease in all the materials. Meanwhile, ABI5 gradually decreases, followed by an increase and another decrease in expression in all materials. Compared to pTRV2::00 plants, the relative expression of ERF1 in pTRV2::*GhAPX7* plants significantly decreased, but the increase was more pronounced (Figure 5e–h).

SOS2 plays a crucial role in the Salt Overly Sensitive (SOS) signaling pathway. In rice, overexpressing SOS2 can enhance drought tolerance [30]. However, exacerbating drought stress increased the fluctuation in SOS2 expression in the pTRV2::00 plants of drought-sensitive materials. On the contrary, increased drought stress initially increased SOS2, followed by a decrease in pTRV2::00 plants of drought-resistant materials, with an overall significant increase. After silencing *GhAPX7*, SOS2 expression showed similar fluctuations in both extreme materials, with minimal expression (Figure 5i,j).

#### 2.2.4. Gene Differential Expression Analysis

A total of 24 samples were sent for transcriptome sequencing (Table 1).

This experiment focused on analyzing the data of A−vs−E, B−vs−F, I−vs−K, and J−vs−L to explore whether the silencing of the *GhAPX7* gene affects the drought response process in cotton. The comparison analysis was conducted between the blank control (pTRV2::00) and experimental groups (pTRV2::*GhAPX7*) of different materials after drought treatment. By analyzing the differential genes related to drought function between the two groups and enriching the pathways related to drought-related differential genes, the function of the *GhAPX7* gene in the drought response process of cotton was further elucidated.

As shown in Table 2, the A−vs−E group had 5023 DEGs, including 2567 (51.10%) up-regulated and 2456 (48.90%) down-regulated genes. The B−vs−F group contained 9485 DEGs, with 4261 (44.92%) up-regulated and 5224 (55.08%) down-regulated genes. In the I−vs−K group, there were 15,615 DEGs, with 7637 (48.91%) up-regulated and 7978 (51.09%) down-regulated genes. In the J−vs−L group, there were 13,754 DEGs, with 6626 (48.18%) up-regulated and 7128 (51.82%) down-regulated genes. Table 1 shows the statistics of the DEGs.

#### 2.2.5. GO Classification Enrichment Analysis

**Figure 6:**
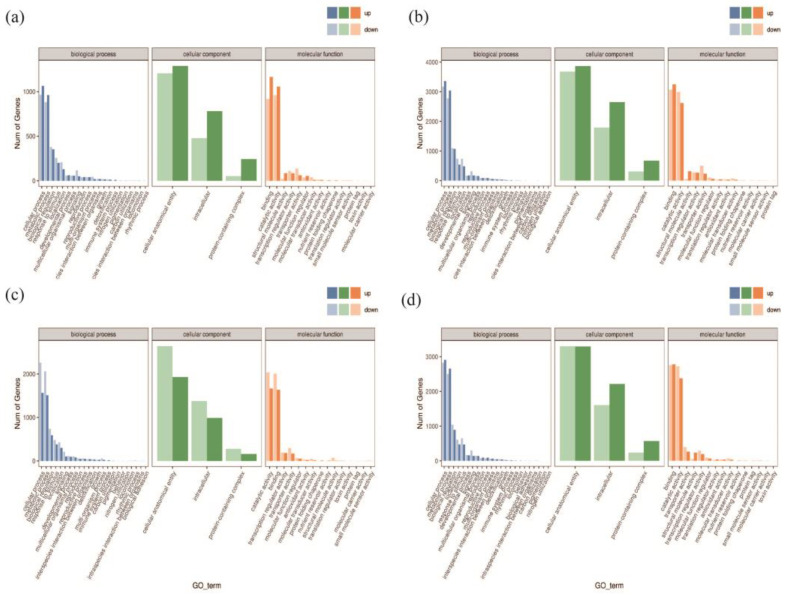
Differentially expressed genes (DEGs) and GO annotation statistics. Note: (**a**) GO annotation classification statistics of DEGs in A−vs−E; (**b**) GO annotation classification statistics of DEGs in I−vs−K; (**c**) GO annotation classification statistics of DEGs in B−vs−F; (**d**) GO annotation classification statistics of DEGs in J−vs−L. Note: the horizontal axis represents the GO categories, the vertical axis represents the number of genes, and different colors represent different primary classifications.

Figure 6 shows the classification results that categorize the DEGs into three main classes: biological processes, cellular components, and molecular functions. In the A−vs−E group, the up-regulated genes in the biological process category mostly enriched the cell regulation system. The up-regulated genes mostly enriched the binding systems in the molecular function category. In contrast, the up-regulated genes in the cellular component category largely enriched the cell structures and biological regulation systems. The A−vs−E and I−vs−K groups share enriched pathways, and all the genes were up-regulated.

Similarly, the A−vs−E and B−vs−F groups share enriched pathways, and all the genes were down-regulated in the B−vs−F group. A−vs−E and J−vs−L also share enriched pathways. In the J−vs−L group, most up-regulated genes belonged to the biological process category and enriched the cell regulation systems. The up-regulated genes mostly enriched the binding systems in the molecular function category. Up-regulated and down-regulated genes enriched cell structures and biological regulation systems in the cellular component category. Therefore, the drought-resistant gene *GhAPX7* up-regulates genes involved in biological regulation in drought-stressed empty pTRV2:00 XLZ26 and KK1543 materials. In contrast, removing *GhAPX7* from the silenced-gene pTRV2::*GhAPX7* XLZ26 and KK1543 materials up-regulated some genes in the same pathways in response to drought stress, thereby reducing the resistance to drought in plants.

**Figure 7:**
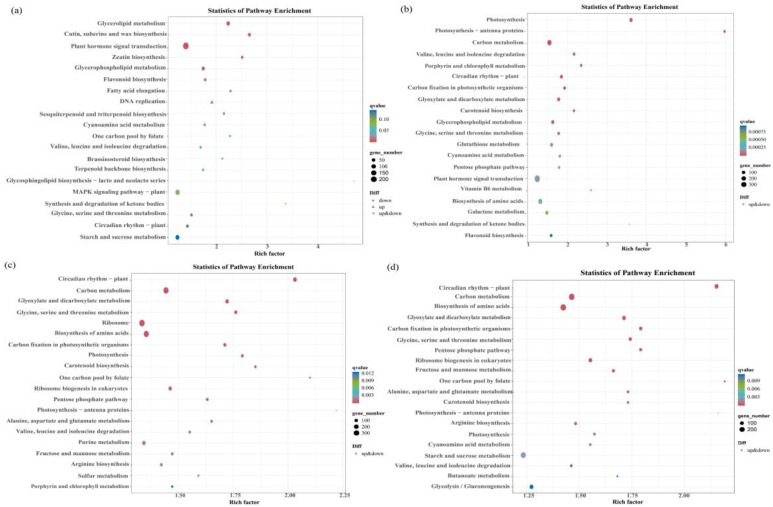
Bubble plots of differentially expressed genes (DEGs) that enrich the KEGG pathways. Note: (**a**) bubble plot of DEGs that enriched the KEGG pathways for A−vs−E; (**b**) bubble plot of DEGs that enriched the KEGG pathways for the I_ vs_ K group; (**c**) bubble plot of DEGs that enrich the KEGG pathways for B−vs−F; and (**d**) bubble plot of DEGs that enrich the KEGG pathways for J_vs _L.

Among the four differentially expressed gene groups (group A−vs−E, group I−vs−K, group B−vs−F, and group J−vs−L), there were more differential genes enriched in the phytohormone signaling pathway; there were 209 (85 up-regulated and 124 down-regulated), 439, (192 up-regulated and 274 down-regulated), 347 (131 up-regulated and 216 down-regulated), and 394 (133 up-regulated and 261 down-regulated) differential genes (Figure 7a–d).

**Figure 8:**
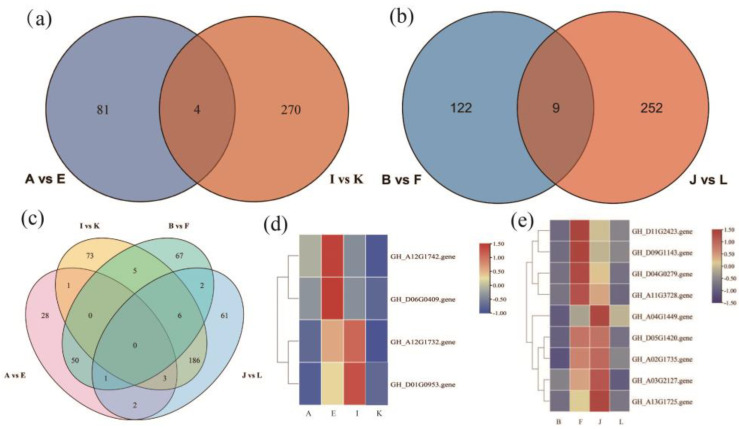
Venn diagrams and heatmaps of differentially expressed genes (DEGs). (**a**) Venn diagram of DEGs between A−vs−E and I−vs−K; (**b**) Venn diagram of DEGs between B−vs−F and J−vs−L; (**c**) Venn diagram of DEGs among A−vs−E, I−vs−K, B−vs−F, and J−vs−L; (**d**) heatmap of the four overlapping genes between A−vs−E and I−vs−K; and (**e**) heatmap of the nine overlapping genes between B−vs−F and J−vs−L.

The KEGG enrichment analysis revealed the plant hormone signaling pathway as the most significantly enriched pathway regulating the drought resistance function of *GhAPX7*. Based on previous results, this study compared the up-regulated genes in the plant hormone signaling pathway of the A−vs−E group and the down-regulated genes of the I−vs−K group. The results identified four common genes between these groups (Figure 8a). Similarly, the up-regulated genes in the plant hormone signaling pathway of the B−vs−F group and the down-regulated genes of the J−vs−L group revealed nine common genes (Figure 8b). Furthermore, the up-regulated genes in the plant hormone signaling pathway of A−vs−E and B−vs−F and the downregulated genes of I_ vs_ K and J_ vs_ L revealed no common genes (Figure 8c). Additionally, Figure 8d shows heatmaps for the four common genes identified (accession numbers GH_A12G1732, GH_A12G1742, GH_D01G0953, and GH_D06G0409) in the A−vs−E and I_ vs_ K groups (Figure 8d), and the nine (accession numbers GH_A02G1735, GH_A03G2127, GH_A04G1449, GH_A11G3728, GH_A13G1725, GH_D04G0279, GH_D05G1420, GH_D09G1143, and GH_D11G2423) in B−vs−F and J−vs−L (Figure 8e).

## 3. Discussion

### 3.1. Reliability of Methods and Designs to Study the Drought Tolerance Function of GhAPX7 in Cotton

The VIGS technique using TRV allows for rapid and efficient verification of gene function. This method improves the efficiency of validating the drought resistance function of *GhAPX7* [31]. In addition, transcriptome sequencing is an important technology for studying gene expression [32], providing a convenient and effective way to explore specific plant genes, control specific traits, understand related metabolic pathways, and explore their molecular mechanisms. In this study, BioMarker’s RNA-seq synthetic sequencing method was used to explore the drought resistance expression mechanism of *GhAPX7* in dryland cotton.

Secondly, the drought-resistant cotton material KK1543 and the drought-sensitive material XLZ26 were selected in this study, on the one hand, in order to illustrate the universality of the drought-tolerant function of *GhAPX7* in different cotton materials, and on the other hand, to allow the results of the two groups of tests to corroborate with each other and increase the reliability of the results.

### 3.2. Effects of Silencing the GhAPX7 Gene on Plant Physiology and Biochemistry

Environmental stressors cause plants to produce excessive ROS, leading to cellular oxidative damage or apoptosis [33]. APX (EC 1.11.1.11) maintains cellular redox homeostasis by scavenging ROS [34]. Studies have shown that *APX* genes are important in plant responses to stress. Plants overexpressing *APX* increase ascorbate peroxidase activity and exhibit enhanced tolerance to salt, drought, and cold stress [35]. The results in Figure 3 indicate that silencing of *GhAPX7* can reduce the water content of cotton leaves, thus affecting the drought resistance of cotton to some extent. Therefore, in the present study, drought stress significantly reduced the APX enzyme activity in pTRV2::*GhAPX7* plants with both XLZ26 and KK1543 as receptor materials (Figure 4g,h). Transgenic poplars with antisense *APX* develop fewer roots, have weaker growth, and longer growth cycles under favorable growing conditions. However, salt treatment induces rapid symptoms of salt damage, increasing H_2_O_2_ and MDA and decreasing ASA (Ascorbic Acid) and total chlorophyll in transgenic compared to wild-type poplars [36]. In the silencing tests of both receptor materials in this study, the physiological and biochemical parameters showed the following patterns. Drought stress significantly reduced the MDA, chlorophyll, and proline content of pTRV2::*GhAPX7* compared to pTRV2::00 plants (Figure 4a,b). In contrast, the chlorophyll content in pTRV2::*GhAPX7* plants with KK1543 as the receptor material decreased (Figure 4e,f). This may be due to the two receptor materials in the regulation of chlorophyll content. There are genetic differences in the genes in the pathway.

### 3.3. Effects of Silencing the GhAPX7 Gene on Transcriptional Expression in Plants

In recent years, domestic and international research on cotton *APX* has mainly focused on cloning specific genes related to cotton fiber and the *APX* regulatory mechanism of cotton fiber development [37]. Additionally, some studies have indicated that introducing exogenous *tAPX* genes enhances the PSⅡ photochemical activity and antioxidant capacity of transgenic cotton [38]. However, research on the molecular mechanisms of other stress resistance avenues is limited, especially for drought resistance in upland cotton. Therefore, studying the molecular mechanism of drought resistance genes in upland cotton is significant.

**Figure A1:**
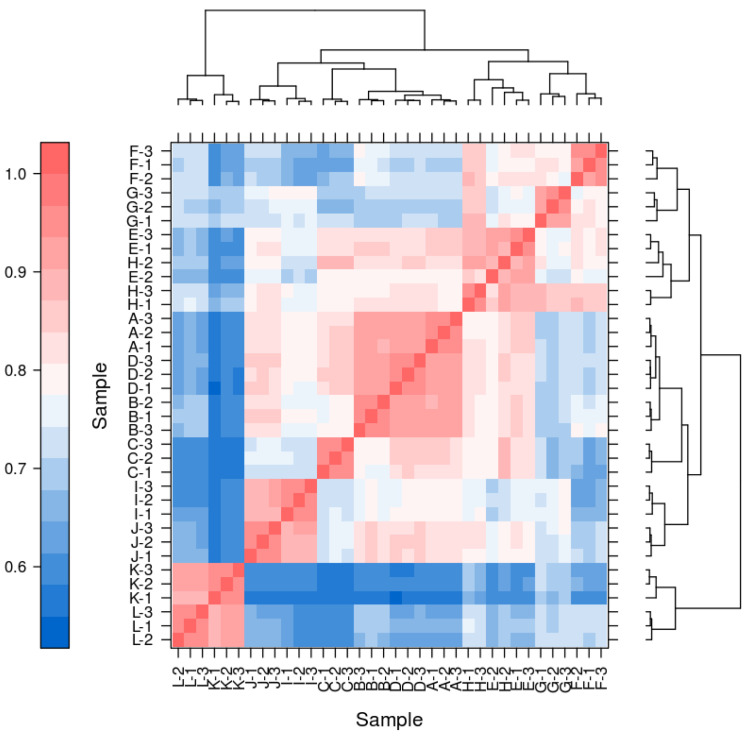
The correlation heatmap of pairwise samples.

**Figure A2:**
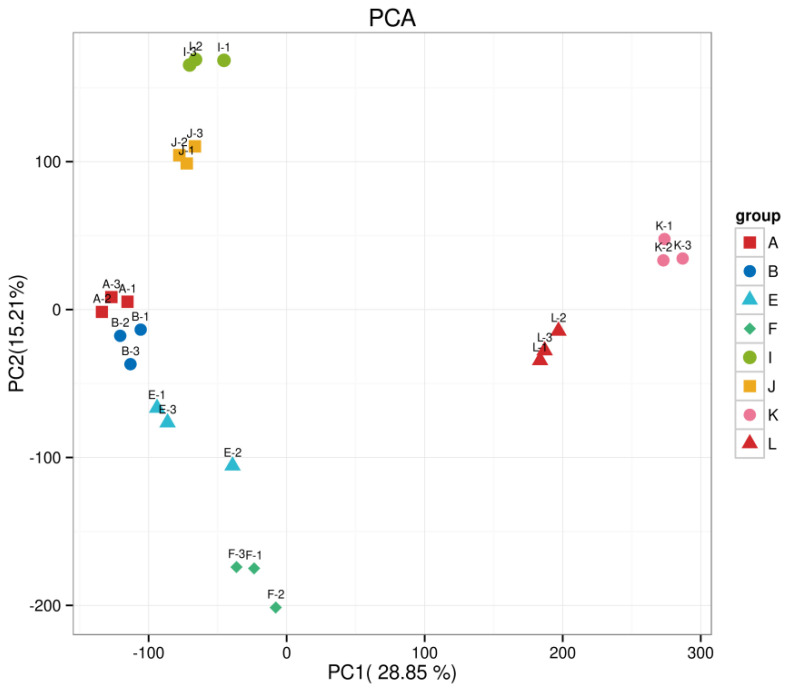
Principal component analysis plot.

In this study, we analyzed the differences in the transcriptional expression of plants after silencing *GhAPX7* in both drought-resistant and drought-sensitive materials and found possible regulatory pathways that *GhAPX7* genes are involved in and different effects generated by the expression of antisensitive materials’ genes (For more details, please refer to Figure A1 and Figure A2 in the Appendix A). In the XLZ 26 material and KK1543 material, after *GhAPX7* silencing, GO analysis (Figure 6a–d) revealed that a common point is the cell component enriched genes, the number of which is around 3900; the difference is (Figure 6c) that in three components, the expression of down-regulated genes is more than the expression of up-regulated genes; Figure 6a,b,d show the opposite. According to the above results, the genes that are speculated to exert drought resistance functions related to *GhAPX7* are concentrated in the cell fraction, and the number of up-regulated genes is higher than the number of down-regulated genes.

In the XLZ 26 material and the KK1543 material, after *GhAPX7* silencing, the KEGG analysis (Figure 7) results are in common; the top three pathways with the largest number of enriched genes were the phytohormone signal transduction pathway, MAPK signaling pathway, plant–pathogen interaction. In the three pathways, the highest number of differentially expressed genes was 435 and the lowest was 237. After the silencing of the drought resistance material and *GhAPX7*, the functional genes were different. It is speculated that the key genes in the drought resistance function of the two extreme materials may have genetic differences.

Two main classes of significantly differentially expressed genes function in the XLZ 26 material and KK1543 material with silenced *GhAPX7*. The first category includes the cytokinin receptor (GH_A12G1732). The most obvious physiological role of cytokinin is to promote cell division and differentiation, delay the degradation of protein and chlorophyll, delay aging, and have the effect of green protection. The second category includes participation in Environmental Information Processing and plant hormone signal transduction, by Brassinoeusterins, (GH_A12G1742), SAUR family protein (GH_D01G0953), transcription factor TGA (GH_D06G0409), ABA response-element-binding factor (GH_A03G2127), transcription factor MYC2 (GH_A02G1735), the ARR family (GH_A04G1449), the DELLA protein (GH_A11G3728), metabolic protein kinase (GH_A13G1725) the GH3 gene family (GH_D04G0279), the ZIM domain protein of jasmonate (GH_D05G1420), the gibberellin receptor GID1 (GH_D09G1143), and xylosyltransferase TCH4 (GH_D11G2423) (Figure 8a,d).

Based on the functions of the above genes (Figure 8d,e), the upstream regulatory *GhAPX7* genes are inferred to be as follows: GH_A12G1732, GH_D01G0953, GH_A04G1449, GH_D05G1420, GH_A02G1735, GH_A03G2127, GH_A13G1725; the downstream regulatory genes are as follows: GH_A12G1742, GH_D06G0409, GH_D11G2423, GH_D09G1143, GH_D09G1143, GH_D04G0279, GH_A11G3728. These results provide some reference for further analyzing the molecular mechanism of *GhAPX7* drought resistance and exploring the key genes in the regulation pathway of drought resistance.

## 4. Materials and Methods

### 4.1. Plant Materials

The test materials included two Xinjiang upland cotton resources, KK1543 (drought-resistant) and Xinluzao 26 (drought-sensitive) [39], preserved by the Crop Genetic Improvement and Germplasm Innovation Key Laboratory at Xinjiang Agricultural University.

### 4.2. Indoor Cotton Cultivation

Indoor cotton cultivation involved treatment with 30% H_2_O_2_ for 2 h and rinsing 2–3 times with water. The cleaned seeds were soaked in water until they turned pale before planting in pots containing a mixture of black soil and vermiculite (2:1). The pots were placed in a dark environment at 25 °C. After cotton seedling emergence (3 days), the pots were transferred to an environment with a photoperiod of 12 h light/12 h dark. Natural drought stress was administered after the cotyledons had fully expanded.

The leaves (same part) of 12 plants each of silent cotton, XLZ26 and KK1543 control TRV2:00, and TRV2:*GhAPX7* were picked and immediately the fresh weight of the leaves (g) was measured; at the end of weighing, the leaves were immediately dried in an oven, killed at 80 degree Celsius for 30 min, and then dried at 50 degree Celsius for 12 h until the leaves were completely dried. Then, the weight of the dried leaves (g) was measured.

### 4.3. RNA Extraction and RT-qPCR Analysis

Drought-resistant KK1543 and drought-sensitive XLZ26 [39] were chosen as extreme materials for the RT-qPCR experiment using key genes. KK1543 and XLZ26 are wild-type plants grown in the soil under greenhouse conditions with a photoperiod (12 h light/12 h dark) at 25 °C. Cotton seedlings were grown until the three-leaf stage before subjection to natural drought stress. Three uniform cotton seedlings were selected for each treatment group. Root, stem, and leaf samples were collected when the soil moisture content reached 50, 40, 30, 20, and 10%. The samples were immediately frozen in liquid nitrogen and stored for subsequent analysis.

Specific primers were designed using DNA Man (LynnonBiosoft, San Ramon, CA, USA) and NCBI (National Center for Biotechnology Information, USA) tools. Total RNA was extracted from the samples using the RNA prep Pure Polysaccharide Polyphenol Plant Total RNA Extraction Kit (Tiangen, Beijing, China), following the manufacturer’s instructions. Next, first-strand cDNA was synthesized using a reverse transcription kit (Tiangen, Beijing, China). Cotton Ubiquitin7 (UBQ7) was the reference gene for normalization. Gene expression was analyzed in triplicate using RT-qPCR on the Applied Biosystems™ 7500 Fast Real-Time PCR System (Applied Biosystems, Waltham, MA, USA). Relative expression was calculated using the 2^−ΔΔCt^ method [40]. Data analysis was performed using GraphPad Prism 10.1.2 for Windows.

### 4.4. VIGS Material Phenotypic and Physiological Index Detection, Real-Time Fluorescence Quantitative PCR Analysis

Fifteen days after injection with pTRV2-CLA (when the newly emerged leaves were completely whitened), the tender true leaves of plants injected with pTRV2 and pTRV2-*GhAPX7* were collected. The fluorescence intensity indicated the silencing efficiency of the whitening gene (CLA), and RT-qPCR evaluated the silencing efficiency of candidate genes. The expression of relevant drought-resistant genes, MDA content, proline content (Pro assay) [41], chlorophyll content [42], and APX enzyme activity [43] was also determined. Additionally, photographic records were taken to document the related phenotypes.

### 4.5. Transcriptome Sequencing Analysis of VIGS Plants

After the appearance of phenotypes in control whitening seedlings, silenced plants were selected and subjected to soil moisture drought stress experiments. Leaf samples were collected before and after stress treatment and stored at −80 °C. Altogether, 24 samples were selected for transcriptome sequencing, including three replicates of pTRV2 control and pTRV2-*GhAPX7* silenced materials (Table 1).

Total RNA was extracted using the RNA prep Pure Polysaccharide Polyphenol Plant Total RNA Extraction Kit (Tiangen, Beijing, China). The quality of total RNA was evaluated using UV absorption and denaturing agarose gel electrophoresis. The cDNA libraries were sequenced on an Illumina HiSeq high-throughput sequencing platform (Illumina, San Diego, CA, USA) using synthetic sequencing (SBS), generating large amounts of high-quality data (provided by Baimeike). The *Gossypium hirsutum* (TM_1_v2.1) genome was the reference for sequence alignment and subsequent analysis. Enrichment analysis was conducted using hypergeometric tests to identify significantly enriched Gene Ontology (GO) terms and Kyoto Encyclopedia of Genes and Genomes (KEGG) pathways in the genetic background. GO enrichment and KEGG pathway analyses of differentially expressed mRNAs were performed using the Database for Annotation, Visualization, and Integrated Discovery (DAVID, version 6.8).

Differential expression analysis was performed using the DESeq2 differential analysis software (for groups with biological replicates) and edgeR (for groups without biological replicates). Genes with a fold change ≥ 2 and a false discovery rate (FDR) < 0.01 were considered differentially expressed. The transcriptome data were processed using TBtools (TBtools-II_windows-x64_2_036) to generate Venn diagrams and heatmap visualization.

### 4.6. Subsection Transcriptome Analysis of the Drought Resistance Mechanism of GhAPX7 Gene

#### Transcriptome Analysis of Cotton *GhAPX7* Gene-Silenced Plants

For *GhAPX7* gene silencing, leaf samples were collected from KK1543 and XLZ26 plants under normal watering conditions and after three days of drought stress. Samples included an empty vector control (pTRV2::00), and *GhAPX7* gene-silenced (pTRV2::*GhAPX7*) materials. Each sample group had three replicates with two treatment conditions: normal watering and natural drought stress until the soil moisture reached approximately 25%. In total, 24 samples were prepared for transcriptome sequencing (see Table 1).

### 4.7. Library Quality

The transcriptome libraries of the 24 samples yielded 226.24 Gb of Clean Data, with each sample reaching 5.75 Gb of Clean Data. The percentage of Q30 bases exceeded 92.7% across all samples. The Clean Reads had 96.78 to 97.94% alignment to the reference genome (TM-1), indicating the suitability of the data for further research.

### 4.8. Correlation of the Transcriptome Data

The results indicate a significant correlation among biological replicate samples in this experiment, while substantial differences exist between samples of different materials and treatments (Table 2).

### 4.9. Principal Component Analysis

The distances between biological replicates in this experiment show consistency among replicates, while samples from different treatments are distinctly separated, indicating differences between the groups. The first two principal components explained 28.85% and 15.21% of the variation, respectively (see Figure A2).

### 4.10. Statistical Analyses Subsection

Specific primers were designed using DNA Man (Lynnon Corporation, Quebec, QC, Canada) and NCBI (National Center for Biotechnology Information, USA) tools. Gene expression was analyzed in triplicate using RT-qPCR on the Applied Biosystems™ 7500 Fast Real-Time PCR System (Applied Biosystems, USA). Relative expression was calculated using the 2^−ΔΔCt^ method [40]. The data were organized statistically using an Excel sheet. Data analysis was performed using two-way analysis of variance (ANOVA). Charts were created using GraphPad Prism 10.1.2 for Windows. Transcriptome data analysis was performed using BMKCloud (www.biocloud.net).

## 5. Conclusions

This study combined VIGS with transcriptome sequencing to preliminarily elucidate the molecular mechanism of the *GhAPX7* gene in drought resistance, an important strategy for the rapid and accurate exploration of key drought-resistant genes in cotton and determining gene molecular mechanisms. The RT-qPCR and VIGS experiments revealed the role of *GhAPX7* in the cotton drought stress response pathways and determined its response to drought stress. This transcriptome sequencing of VIGS materials provides an important scientific basis for the preliminary analysis of the regulatory mechanism of involvement of *GhAPX7* in the cotton drought stress response. Moreover, it offers potential strategies for further improving the stress adaptability and yield of cotton.

## Figures and Tables

**Table 1:** Sample names and biological repeats for the transcriptome analysis.

Serial Number	Sample Name	Analytical Sample Name	Biological Repeat Group Numbering
1	0d26V2	A-1	A
2	0d26V2	A-2	A
3	0d26V2	A-3	A
4	0dKKV2	B-1	B
5	0dKKV2	B-2	B
6	0dKKV2	B-3	B
7	0d26A	I-1	I
8	0d26A	I-2	I
9	0d26A	I-3	I
10	0dKKA	J-1	J
11	0dKKA	J-2	J
12	0dKKA	J-3	J
13	Dr26V2	E-1	E
14	Dr26V2	E-2	E
15	Dr26V2	E-3	E
16	DrKKV2	F-1	F
17	DrKKV2	F-2	F
18	DrKKV2	F-3	F
19	Dr26A	K-1	K
20	Dr26A	K-2	K
21	Dr26A	K-3	K
22	DrKKA	L-1	L
23	DrKKA	L-2	L
24	DrKKA	L-3	L

Note: “0d” and “Dr” represent normal watering and drought stress treatments, respectively. “26” and “KK” are Xinluzao 26 and KK1543 materials, respectively. The “A” in the sample name stands for the *GhAPX7* gene. Groups A, B, I, and J represent the empty pTRV2::00 material under normal watering, the KK1543 empty pTRV2::00 material under normal watering, the silenced-gene pTRV2::*GhAPX7* material under normal watering, and the KK1543 silenced-gene pTRV2::*GhAPX7* under normal watering, respectively. Groups E, F, K, and L represent the empty pTRV2::00 material of Xinluzao 26after drought stress, the KK1543 unloaded pTRV2::00 material after drought stress, the Xinluzao26 silenced-gene pTRV2::*GhAPX7* material after drought stress, and the KK1543 silenced-gene pTRV2::*GhAPX7* material after drought stress, respectively.

**Table 2:** Summary of differential gene expression counts.

DEG Set	DEG Number	Up-Regulated	Dowm-Regulated
A−vs−E	5023	2567	2456
B−vs−F	9485	4261	5224
I−vs−K	15,615	7637	7978
J−vs−L	13,754	6626	7128

Note: DEG Set: name of the differentially expressed gene set; DEG Number: number of differentially expressed genes; up-regulated: number of up-regulated genes; and down-regulated: number of down-regulated genes.

## Data Availability

For related data, please visit https://www.ncbi.nlm.nih.gov/sra/PRJNA1124177 (accessed on 14 July 2024).
